# Distinct functional roles for the two SLX4 ubiquitin-binding UBZ domains mutated in Fanconi anemia

**DOI:** 10.1242/jcs.146167

**Published:** 2014-07-01

**Authors:** Christophe Lachaud, Dennis Castor, Karolina Hain, Ivan Muñoz, Jamie Wilson, Thomas J. MacArtney, Detlev Schindler, John Rouse

**Affiliations:** 1 MRC Protein Phosphorylation and Ubiquitylation Unit, College of Life Sciences, Sir James Black Centre, University of Dundee, Dundee DD1 5EH, UK; 2 Department of Human Genetics, University of Wuerzburg, Biozentrum, Wuerzburg, Germany

**Keywords:** SLX4, FANCP, Ubiquitin, Fanconi anemia, UBZ, ICL

## Abstract

Defects in SLX4, a scaffold for DNA repair nucleases, result in Fanconi anemia (FA), due to the defective repair of inter-strand DNA crosslinks (ICLs). Some FA patients have an SLX4 deletion removing two tandem UBZ4-type ubiquitin-binding domains that are implicated in protein recruitment to sites of DNA damage. Here, we show that human SLX4 is recruited to sites of ICL induction but that the UBZ-deleted form of SLX4 in cells from FA patients is not. SLX4 recruitment does not require either the ubiquitylation of FANCD2 or the E3 ligases RNF8, RAD18 and BRCA1. We show that the first (UBZ-1) but not the second UBZ domain of SLX4 binds to ubiquitin polymers, with a preference for K63-linked chains. Furthermore, UBZ-1 is required for SLX4 recruitment to ICL sites and for efficient ICL repair in murine fibroblasts. The SLX4 UBZ-2 domain does not bind to ubiquitin *in vitro* or contribute to ICL repair, but it is required for the resolution of Holliday junctions *in vivo*. These data shed light on SLX4 recruitment, and they point to the existence of currently unidentified ubiquitylated ligands and E3 ligases that are crucial for ICL repair.

## INTRODUCTION

**Fig. 1.**
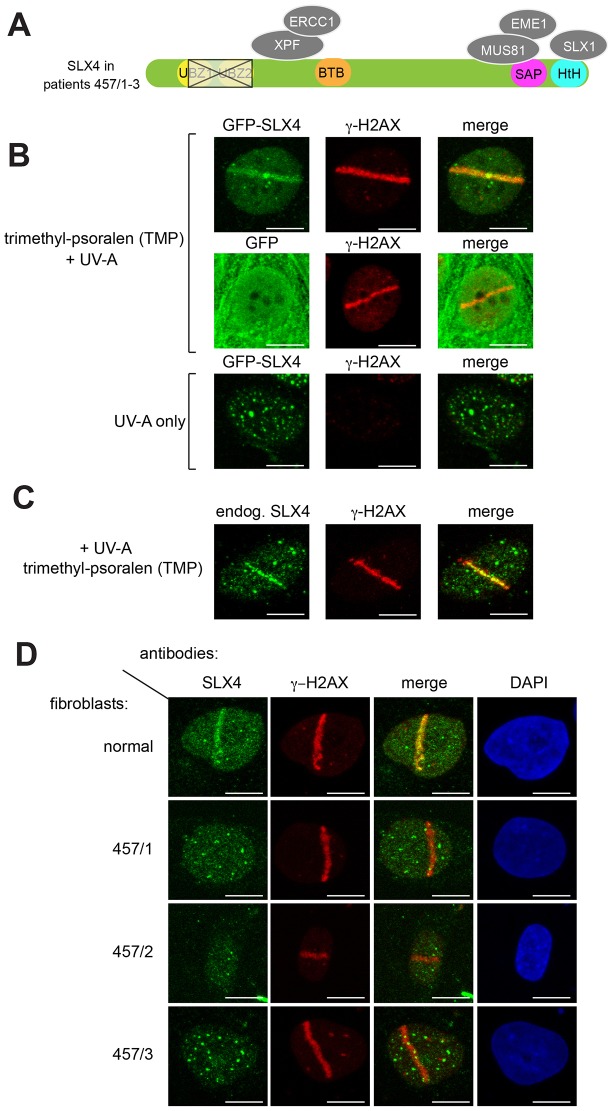
**Recruitment of SLX4 to sites of localized DNA damage induced by PUVA.** (A) Diagram showing SLX4 domain organization and associated nucleases. Siblings 457/1, 457/2 and 457/3 are FA patients with a deletion (outlined in black) removing all of the second of the tandem SLX4 UBZ domains and part of the first. BTB; Broad-complex, Tramtrack, Bric-a-brac domain: SAP; SAF-A/B, Acinus and PIAS motif: HtH; helix-turn-helix motif. (B) U2OS cells stably expressing GFP–SLX4 (upper and lower panels) or GFP only (middle panels) were incubated (or not) with trimethyl-psoralen (TMP; 20 µM, 60 min) and subjected to subnuclear micro-irradiation using a 355-nm UV-A laser. Cells were fixed and subjected to indirect immunofluorescence analysis with antibodies against GFP or γ-H2AX. (C) As for B except the localization of endogenous SLX4 (endog.) in U2OS cells was examined. (D) Cells from FA patients 457/1, 457/2 and 457/3 or normal human fibroblasts were treated as in B and endogenous SLX4 localization was analyzed by using indirect immunofluorescence. Scale bars: 10 µm.

Human SLX4 is a scaffold that coordinates the structure-selective nucleases XPF–ERCC1, MUS81–EME1 and SLX1 (Fekairi et al., 2009; Muñoz et al., 2009; Svendsen et al., 2009), which together can cleave branched DNA repair intermediates. SLX1 and MUS81–EME1, for example, act together to resolve Holliday junctions that escape dissolution by the BLM–TOP3–RMI1–RMI2 (BTR) complex; these junctions are thought to arise during repair of collapsed replication forks. The tethering of MUS81 and SLX1 to the SLX4 scaffold coordinates the dual incisions, made by the two nucleases, that are required for Holliday junction resolution (Castor et al., 2013; Wyatt et al., 2013). SLX4, together with associated nucleases, is important for the repair of DNA inter-strand crosslinks (ICLs) and, consequently, bi-allelic mutations in SLX4 (also known as FANCP) cause Fanconi anemia (FA) (Kim et al., 2011; Stoepker et al., 2011), a rare condition characterized by developmental defects, bone marrow failure and cancer predisposition (Moldovan and D'Andrea, 2009). We previously described three German siblings with FA (patients 457/1–3) caused by a small deletion in SLX4 that removes all of the second and most of the first of two tandem UBZ4-type ubiquitin-binding domains (Fig. 1A) (Stoepker et al., 2011). Therefore, one or both of the UBZ domains are vital for SLX4 function in ICL repair.

UBZ4-type domains have been implicated in recruiting proteins such as FAN1 to sites of DNA damage (Hofmann, 2009). SLX4 recruitment to sub-nuclear foci in mitomycin-C-treated DT-40 cells was reported to require both the single UBZ domain in chicken SLX4 and mono-ubiquitylation of FANCD2 (Yamamoto et al., 2011). The latter is a central component of the FA pathway that is mono-ubiquitylated by the FA core complex at Lys561 in response to ICLs (Garcia-Higuera et al., 2001; Taniguchi et al., 2002). Mono-ubiquitylated FANCD2 recruits the FAN1 nuclease to sites of DNA damage through a UBZ4 domain in human cells but it is not yet clear whether this also applies to SLX4. Although the recruitment of proteins such as FANCD2 can be visualized by the formation of sub-nuclear foci at the sites of DNA damage, this visualization is difficult for SLX4 because it forms foci (corresponding to telomeres) even in the absence of DNA damage (Wilson et al., 2013).

It is not clear why mammalian SLX4 has two UBZ domains whereas SLX4 homologs in some other eukaryotes have only one. It is not known whether one or both of the UBZ domains in human SLX4 are required for recruitment of the protein to sites of DNA damage, for DNA repair and/or for Holliday junction resolution. Furthermore, it is not yet clear whether defective recruitment of SLX4 in FA cells expressing UBZ-deleted SLX4 explains the ICL repair defects in these cells. In this study, we address these issues.

## RESULTS AND DISCUSSION

### SLX4 containing UBZ deletion fails to localize to sites of ICL induction in FA cells

We tested the ability of human SLX4 to localize along a track of ICLs induced by psoralen conjugates (Thazhathveetil et al., 2007). Human U2OS cells stably expressing GFP–SLX4 were exposed to trimethyl-psoralen (TMP) and micro-irradiated along a track in cell nuclei using a 355-nm UV-A laser. As shown in Fig. 1B, GFP–SLX4 (upper panels), but not GFP (middle panels), formed a stripe along the laser track, which also contained the phosphorylated histone variant γ-H2AX; no stripe was formed when cells had not been exposed to TMP (Fig. 1B, lower panels). Indirect immunofluorescence analysis with antibodies raised in-house (Wilson et al., 2013) revealed that endogenous SLX4 was also recruited to sites of psoralen–UVA laser (PUVA)-induced DNA damage (Fig. 1C). Every cell that had a stripe of γ-H2AX also displayed an SLX4 stripe (data not shown).

Next, we tested the role of the SLX4 UBZ domains in recruitment to sites of ICLs. Whereas SLX4 in normal human fibroblasts formed stripes along tracks of PUVA-induced DNA damage, SLX4 in fibroblasts from patients 457/1, 457/2 and 457/3 did not (Fig. 1D). By contrast, immunoblotting of SLX4 immunoprecipitates showed that SLX4 was expressed at close-to-normal levels in fibroblasts from FA patients 457/1–3, and that it interacted normally with XPF–ERCC1, MUS81–EME1, SLX1 and C20ORF94 (supplementary material Fig. S1A,B). These data suggest that at least one of the two SLX4 UBZ domains recognizes a ubiquitylated protein at DNA damage sites, leading to SLX4 recruitment.

### Only one of the two SLX4 UBZ domains binds to ubiquitin polymers

**Fig. 2.**
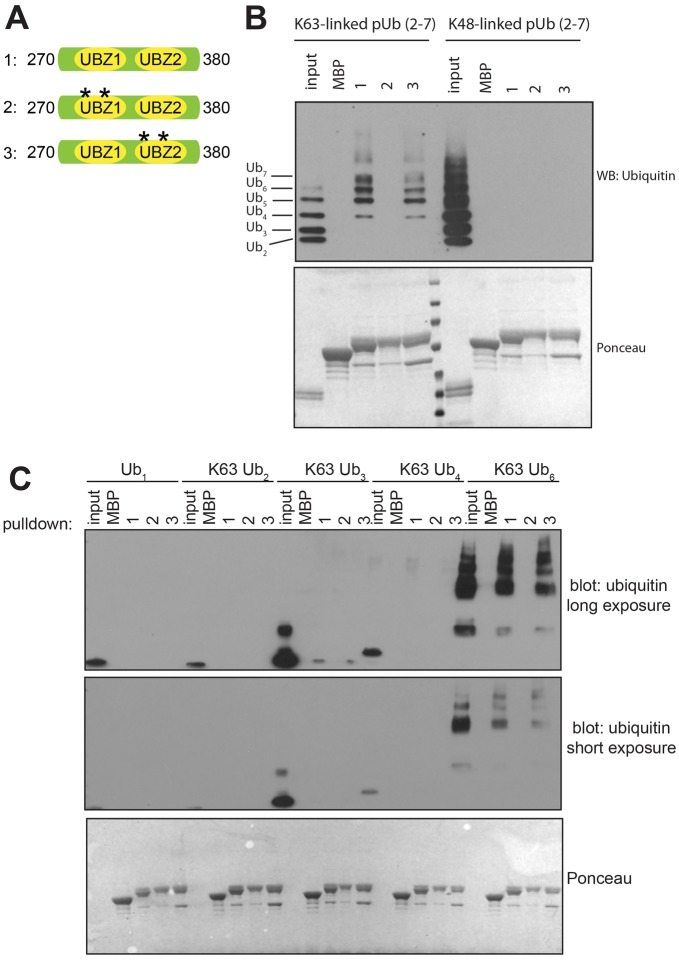
**Testing the ability of the SLX4 UBZ domains to bind to ubiquitin.** (A) Diagram of the MBP-tagged SLX4 fragments used in ubiquitin-binding assays. Asterisks denote cysteine to alanine mutations at Cys 296 and Cys 299 in UBZ-1 (fragment 2) or Cys 336 and Cys 339 in UBZ-2 (fragment 3). (B) The fragments shown in A or MBP alone were immobilized on amylose–agarose and incubated with K48-linked or K63-linked poly-ubiquitin (pUb, 2–7) chains. Pulldowns were subjected to SDS-PAGE and immunoblotted (WB) with anti-ubiquitin antibodies. The bottom panel shows a Ponceau staining of the membrane performed prior to blotting. (C) Same as for B, except that mono-ubiquitin or K63-linked poly-ubiquitin chains of the indicated length were used with fragments 1–3.

We next wished to explore the relative contribution of the individual UBZ domains to SLX4 function, and we first investigated ubiquitin binding. Maltose-binding protein (MBP)-fused fragments of SLX4 containing both UBZ domains (UBZ-1+UBZ-2) were immobilized on amylose–agarose beads. Mutant proteins in which conserved cysteine residues in either the first or the second UBZ domain were mutated to alanine – C296A+C299A (UBZ-1*) and C336A+C339A (UBZ-2*), respectively – were used as controls (Fig. 2A). MBP-tagged SLX4 (UBZ-1+UBZ-2) showed robust binding to K63-linked poly-ubiquitin chains but not to K48-linked polymers (Fig. 2B). Mutating UBZ-1 abolished binding to K63-linked poly-ubiquitin, whereas mutating UBZ-2 had no effect. Similar data were obtained when the MBP tag was replaced with GST (supplementary material Fig. S2A,B). Consistent with these data, GST–UBZ-1 alone bound to K63-linked poly-ubiquitin, and mutating the conserved cysteine residues abolished binding, whereas GST–UBZ-2 alone did not bind to ubiquitin (supplementary material Fig. S2A,B). A minimum of six ubiquitin moieties was required for SLX4 (UBZ-1+UBZ-2) fused to MBP (Fig. 2C) or GST (supplementary material Fig. S2C) to bind to ubiquitin chains. Binding to mono-, di-, tri- or tetra-ubiquitin was much weaker. These data show that SLX4 domain UBZ-1, but not UBZ-2, binds to poly-ubiquitin chains with a preference for K63- over K48-linkages.

### Recruitment of SLX4 to sites of DNA damage requires the UBZ-1 domain, but not the UBZ-2 domain, FANCD2, RNF8, RAD18 or BRCA1

**Fig. 3.**
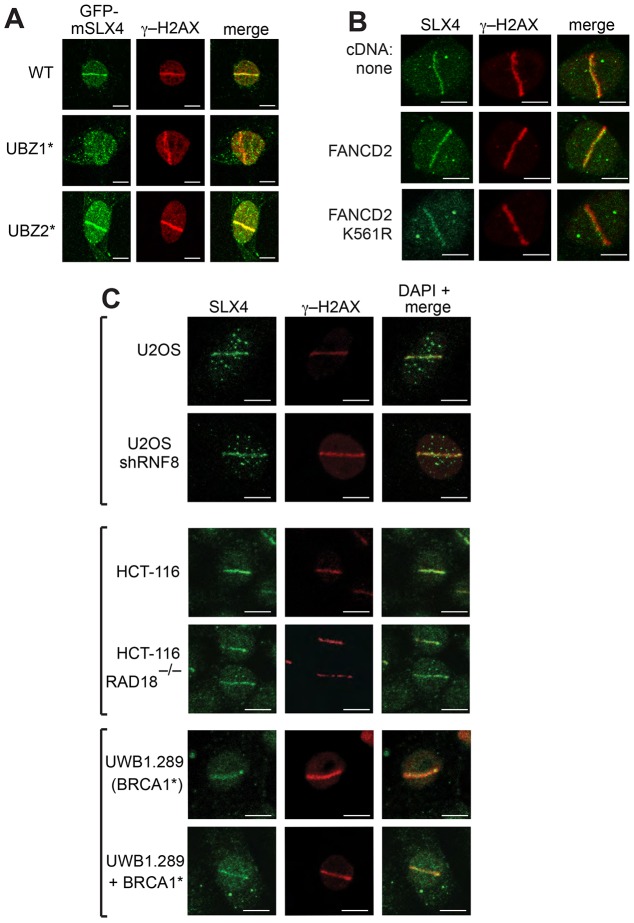
**Relative contributions of the SLX4 UBZ domains to DNA repair and recruitment to DNA damage sites.** (A) *Slx4*^−/−^ MEFs were infected with retroviruses expressing GFP-tagged mouse (m)SLX4 wild-type (WT), SLX4 UBZ-1* or SLX4 UBZ-2*. Cells were incubated with trimethyl-psoralen (TMP) and subjected to subnuclear micro-irradiation using a 355-nm UV-A laser. Cells were fixed and subjected to indirect immunofluorescence analysis with antibodies against GFP or γ-H2AX. (B) PD20 cells lacking FANCD2 (upper panels), or PD20 cells stably expressing FANCD2 (middle panels) or FANCD2 K561R (lower panels), were subjected to laser micro-irradiation as in A. Endogenous SLX4 and γ-H2AX were analyzed. (C) Same as for B, except that U2OS cells stably expressing RNF8 shRNA plus parental cells, HCT-116 cells lacking RAD18 plus parental cells or UWB1.289 cells lacking BRCA1 were used. UWB1.289 cells expressing BRCA1 were used as a control. For each population, ∼300 cells were counted, and representative images are shown. Scale bars: 10 µm.

We next tested the effect of UBZ mutations on the localization of mouse SLX4 expressed in murine embryonic fibroblasts (MEFs) from *Slx4*^−/−^ mice (Castor et al., 2013). *Slx4*^−/−^ MEFs were infected with viruses containing wild-type SLX4 fused to GFP or SLX4 bearing mutations in UBZ-1 (UBZ-1*) or UBZ-2 (UBZ-2*). Both wild-type SLX4 and SLX4-UBZ-2* were recruited to the sites of PUVA-induced ICLs, but the SLX4-UBZ-1* mutant was not recruited to sites of DNA damage in any of the cells in the population that had been micro-irradiated (Fig. 3A; data not shown). Consistent with these data, UBZ-1 alone fused to both a nuclear localization signal and GFP was capable of localizing at sites of PUVA-induced DNA damage, similar to wild-type SLX4, although on average the intensity of the stripes was weaker than that of stripes observed in cells containing wild-type SLX4 (supplementary material Fig. S3).

Mono-ubiquitylation of FANCD2 was reported to mediate recruitment of chicken SLX4 to subnuclear foci in DT-40 cells (Yamamoto et al., 2011). These data are at odds, however, with the preference of the human SLX4 UBZ-1 domain for ubiquitin polymers over mono-ubiquitin (Fig. 2C). In this light, we found that SLX4 could form normal stripes along tracks of DNA damage induced by PUVA treatment in FANCD2^−/−^ (PD20) human cells transfected with empty vector, in a manner that was indistinguishable from PD20 cells expressing wild-type FANCD2 or a mutant K516R FANCD2 that could not be ubiquitylated (Fig. 3B) (Garcia-Higuera et al., 2001).

Besides the FA core complex that ubiquitylates FANCD2 and FANCI, several other E3 ligases have been implicated in ICL repair, including RAD18, which promotes FANCD2 ubiquitylation (Geng et al., 2010; Song et al., 2010), RNF8, which promotes histone ubiquitylation and recruitment of FAAP20 (Leung et al., 2012; Yan et al., 2012), and BRCA1, although the E3 ligase activity of this protein might not be required for DNA repair (Aressy and Greenberg, 2012). SLX4 recruitment to sites of PUVA-induced damage in (1) U2OS cells stably expressing an RNF8 shRNA that prevented 53BP1 recruitment (supplementary material Fig. S3), (2) HCT-116 cells in which RAD18 was disrupted and (3) UWB1.289 cells, which are null for BRCA1, was indistinguishable from that of the relevant parental control cells; every cell with a stripe of γ-H2AX also had an SLX4 stripe of normal intensity (Fig. 3C). Taken together, these data show that SLX4 is recruited to the sites of DNA damage through its UBZ-1 domain, but the ubiquitylated ligand in question is not FANCD2 in human cells and recruitment does not require the E3 ligases that have already been implicated in ICL repair.

### SLX4 domain UBZ-1, but not UBZ-2, is required for ICL repair

**Fig. 4.**
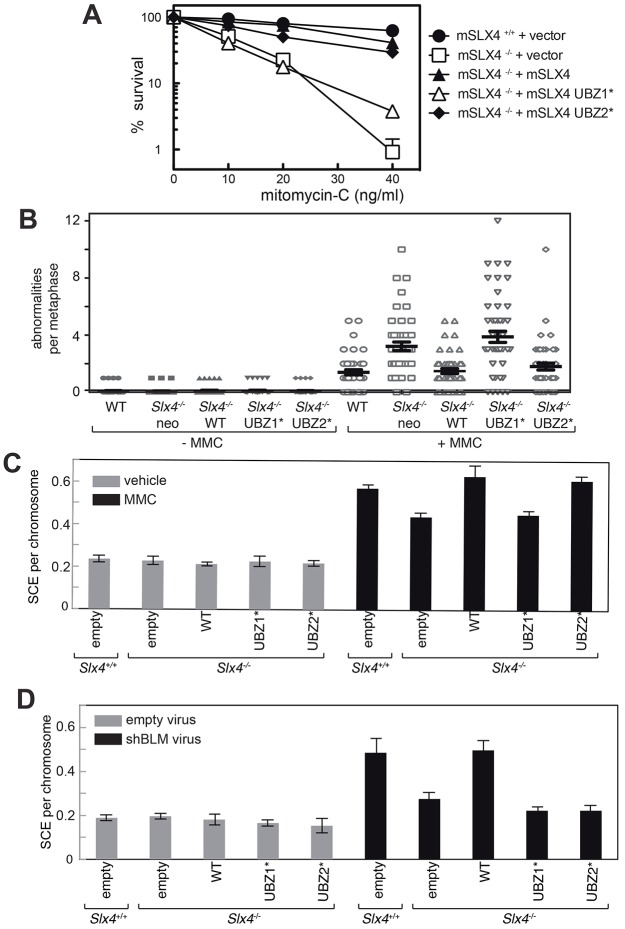
**Relative contributions of the SLX4 UBZ domains to the resolution of Holliday junctions.** (A) Clonogenic survival analysis of *Slx4*^−/−^ MEFs stably expressing untagged mouse (m)SLX4, SLX4 UBZ-1* or SLX4 UBZ-2*, exposed to the indicated doses of MMC. For each genotype, the viability of untreated cells was defined as 100%. Wild-type MEFs and *Slx4*^−/−^ MEFs infected with empty virus (+vector) were used as controls. Data represent the mean±s.e.m., *n* = 3. (B) Metaphase spreads of MEFs treated with MMC (20 ng/ml) were stained with DAPI and analyzed for the presence of radial and broken chromosomes. Each cell line was either treated with MMC or left untreated, and 50 metaphase spreads were analyzed for each condition to determine the number of abnormalities per metaphase. WT, wild-type. Data show the mean±s.d. (C) Wild-type (*Slx4*^+/+^) MEFs, *Slx4*^−/−^ MEFs and *Slx4*^−/−^ MEFs infected with retroviruses expressing wild-type SLX4 (WT) or SLX4 bearing mutations in domains UBZ-1 (UBZ-1*) or UBZ-2 (UBZ-2*) were exposed to MMC (10 ng/ml) and the frequency of SCEs was measured. (D) Same as for C, expect that SCE frequencies in MEFs were measured after the depletion of BLM. In C and D, 400 chromosomes in three metaphase spreads (1200 in total) were analyzed. Data represent the mean±s.e.m.

We next tested the effect of UBZ domain mutations on the sensitivity of cells to mitomycin C (MMC), as a readout of ICL repair. *Slx4*^−/−^ cells infected with empty pBABE-puro vector were much more sensitive to MMC than were wild-type cells infected with the same virus (Fig. 4A). Furthermore, the MMC hypersensitivity of *Slx4*^−/−^ cells was rescued by untagged wild-type mouse SLX4. The SLX4 UBZ-1* mutant was barely capable of rescuing the MMC sensitivity of *Slx4*^−/−^ cells, but the UBZ-2 mutant behaved like wild-type SLX4 (Fig. 4A). Consistent with these data, *Slx4*^−/−^ MEFs showed an increased incidence of chromosome abnormalities (defined as breaks and radial chromosomes), which was rescued by wild-type SLX4. Mutating domain UBZ-1 completely prevented rescue, whereas mutating UBZ-2 made no difference (Fig. 4B). Taken together, these data show that SLX4 domain UBZ-1, but not UBZ-2, is vital for the repair of ICLs.

### Both SLX4 UBZ domains are required for the resolution of Holliday junctions

SLX4 is required for around one third of the sister chromatid exchanges (SCEs) induced by MMC in MEFs (Castor et al., 2013), and we next tested the effect of UBZ domain mutations on the occurrence of SCEs. As shown in Fig. 4C, MMC raises the frequency of SCEs in wild-type MEFs by around 2.5 fold. Compared with wild-type MEFs, *Slx4*^−/−^ MEFs showed ∼30% fewer SCEs after treatment with MMC, but this defect was rescued by wild-type SLX4 or SLX4-UBZ2*. No such rescue was observed with the version of SLX4 bearing mutations in UBZ-1 (Fig. 4C).

SLX4 is required for the nucleolytic resolution of Holliday junctions that escape dissolution by the BTR complex (Castor et al., 2013; Wyatt et al., 2013). We next examined the requirement of the SLX4 UBZ domains for resolution of Holliday junctions in this context. Short hairpin (sh)RNA-expressing retroviruses were used to deplete BLM from *Slx4*^−/−^ MEFs (Castor et al., 2013) (supplementary material Fig. S4). Depletion of BLM from wild-type MEFs caused an increase of ∼2.5 fold in SCE frequency but this increase was severely blunted in *Slx4*^−/−^ MEFs (Fig. 4D). Wild-type SLX4 rescued this defect, but mutating either the first or the second UBZ domain completely prevented rescue (Fig. 4D). These data suggest that both of the UBZ domains in SLX4 are required for the resolution of Holliday junctions that escape dissolution.

In this study, we showed that the mutated form of SLX4 expressed in the cells of German siblings 457/1–3, which lacks all of the second UBZ domain and part of the first, is not recruited to sites of DNA damage. By analyzing the two domains individually, we found that UBZ-1, but not UBZ-2, binds to ubiquitin polymers, mediates recruitment of SLX4 to sites of PUVA-induced DNA damage and is essential for both efficient ICL repair and SCEs induced by ICLs in MEFs. Therefore, proper targeting of SLX4, mediated by UBZ-1, appears to be essential for ICL repair, and it is likely that failure to correctly target SLX4 explains the FA phenotype in patients 457/1-3.

What is the ubiquitylated ligand at sites of DNA damage that recruits SLX4 through UBZ-1? The mono-ubiquitylated form of FANCD2 was reported to be required for the formation of foci in DT-40 chicken cells exposed to MMC (Yamamoto et al., 2011). In the present study, we found that the recruitment of SLX4 to sites of DNA damage induced in this manner occurs normally in cells that lack FANCD2 and in cells lacking RAD18, which is required for FANCD2 ubiquitylation (Geng et al., 2010; Song et al., 2010). These data are consistent with our observation that SLX4 only binds to poly-ubiquitin chains of at least six ubiquitin moieties, whereas FANCD2 is mono-ubiquitylated, not poly-ubiquitylated. We predict that there exists a poly-ubiquitylated ligand that recruits SLX4 to sites of ICLs. It will be interesting to identify this protein as well as the relevant E3 ligase involved, which appears to be distinct from any of the E3 ligases previously implicated in ICL repair.

Although SLX4 UBZ-2 is dispensable for ICL repair, it is required, together with UBZ-1, for the resolution of Holliday junctions that escape dissolution by the BTR complex. Recent data from our lab suggests that the role of the SLX4 complex in repairing ICLs is independent of Holliday junction cleavage and probably reflects the cleavage of alternative branched DNA repair intermediates (Castor et al., 2013). The observation in the present study that SLX4 UBZ-2 is required for the resolution of Holliday junctions that escape dissolution by BTR, but not for ICL repair, is consistent with this idea. Even though UBZ-2 appears to have all of the residues necessary for ubiquitin binding, it does not bind to mono-ubiquitin or to K48- or K63-linked poly-ubiquitin chains *in vitro* (Fig. 2). It is possible, however, that this domain binds to atypical poly-ubiquitin chains such as K11, K27 or K33 linkages or to a ubiquitin-like domain in the protein that recruits SLX4 to sites of DNA damage. Finding the ligands for both of the SLX4 UBZ domains will be an interesting area of investigation.

## MATERIALS AND METHODS

### Cell lines

Mouse embryonic fibroblasts (MEFs) were grown in DMEM supplemented with 10% fetal bovine serum (FBS) supplemented with penicillin [1% (w/v)], streptomycin [1% (w/v)], sodium pyruvate and non-essential amino acids at 37°C in a humidified atmosphere under 5% CO_2_. Normal human fibroblasts or fibroblasts from FA patients 457/1, 457/2 and 457/3 were immortalized with SV-40 and cultured in DMEM medium supplemented with 10% FBS, 1% penicillin-streptomycin and 2 mM glutamine.

### Laser irradiation and confocal microscopy

Cells seeded in 35-mm glass-bottomed dishes were incubated with trimethyl-psoralen [TMP (25 µM); Sigma-Aldrich] for 60 min. A 355-nm UV-A laser attached to a PALM microscope (Zeiss) was used to irradiate a track along cell nuclei. The power of the laser (in terms of percent intensity) was set to 20% to generate ICLs, and the areas were struck at low speed. A 3×20 pixel region internal to the nuclei of the cells was targeted by using a Plan Fluor 40×/1.25 NA oil objective. Cells were then subjected to indirect immunofluorescence. Each experiment was performed a minimum of three times, and a minimum of 100 cells were PUVA treated per replicate.

### Miscellaneous

Indirect immunofluorescence was carried out as described previously (Wilson et al., 2013). Antibodies against mouse SLX4 and analysis of SCE frequencies and chromosome abnormalities were described previously (Castor et al., 2013).

## Supplementary Material

Supplementary Material
