# Supplementary material for: Distinct functional roles for the two SLX4 ubiquitin-binding UBZ domains mutated in Fanconi anemia
Source: J Cell Sci. 2014 Jul 1;127(13):2811–7. doi: 10.1242/jcs.146167 (PMC4075355; doi:10.1242/jcs.146167)
Supplement: Supplementary Material [file supp_127_13_2811__index.html]

Distinct functional roles for the two SLX4 ubiquitin-binding UBZ domains mutated in Fanconi anemia — Supplementary Material 

# Distinct functional roles for the two SLX4 ubiquitin-binding UBZ domains mutated in Fanconi anemia

## JCS146167 Supplementary Material

**Files in this Data Supplement:**

- **Supplementary Material**
