# Supplementary material for: Distinct functional roles for the two SLX4 ubiquitin-binding UBZ domains mutated in Fanconi anemia
Source: J Cell Sci. 2014 Jul 1;127(13):2811–7. doi: 10.1242/jcs.146167 (PMC4075355; doi:10.1242/jcs.146167)
Supplement: Supplementary Material [file supp_127.13.2811_JCS146167.pdf]

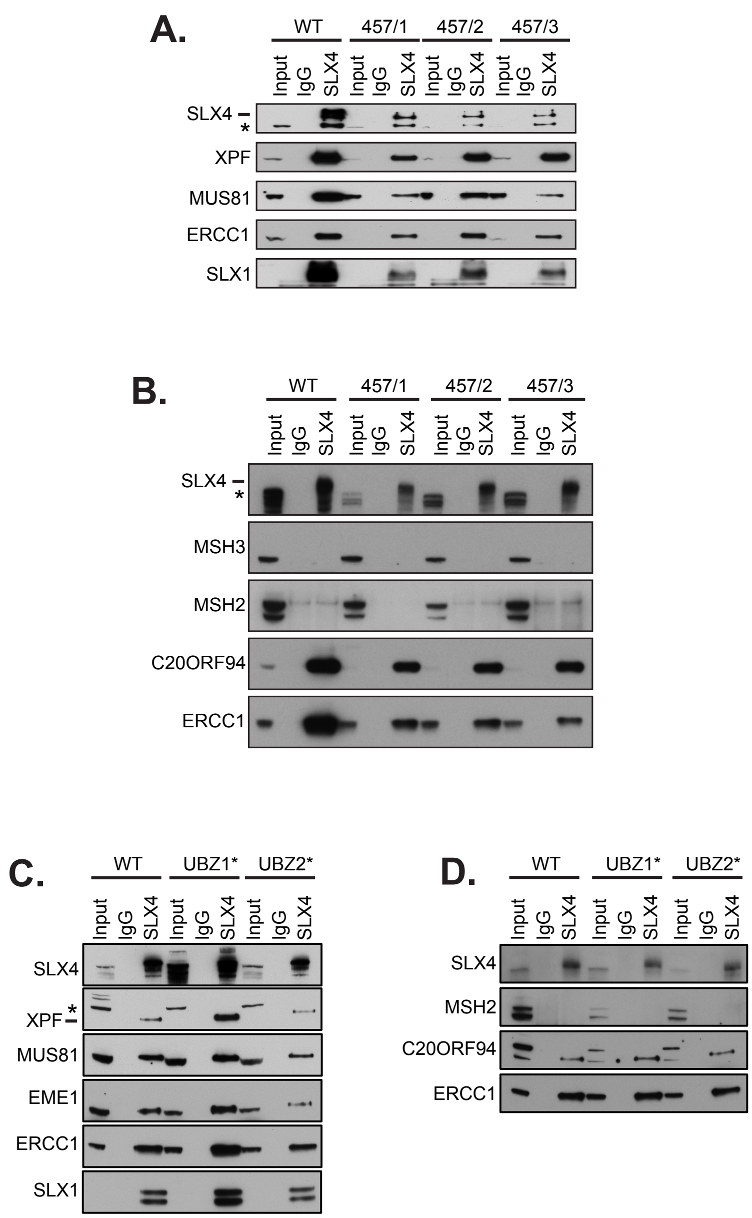

Figure S1. The UBZ-deleted form of SLX4 in FA cells interacts normally with partner proteins

(**A, B**) SLX4 was immunoprecipitated from normal human fibroblasts, or from fibroblasts from FA patients 457/1, 457/2 and 457/3. Non-specific IgG (GFP) was used as control. Precipitates were subjected to immunoblotting with the antibodies indicated. Asterisk indicates non-specific band in cell extracts. (**C, D**) Same as above but using *Slx4*$^{-/-}$ MEFs infected with retroviruses expressing untagged mouse (m) SLX4 wild-type (WT), SLX4 UBZ-1* or SLX4 UBZ-2*.

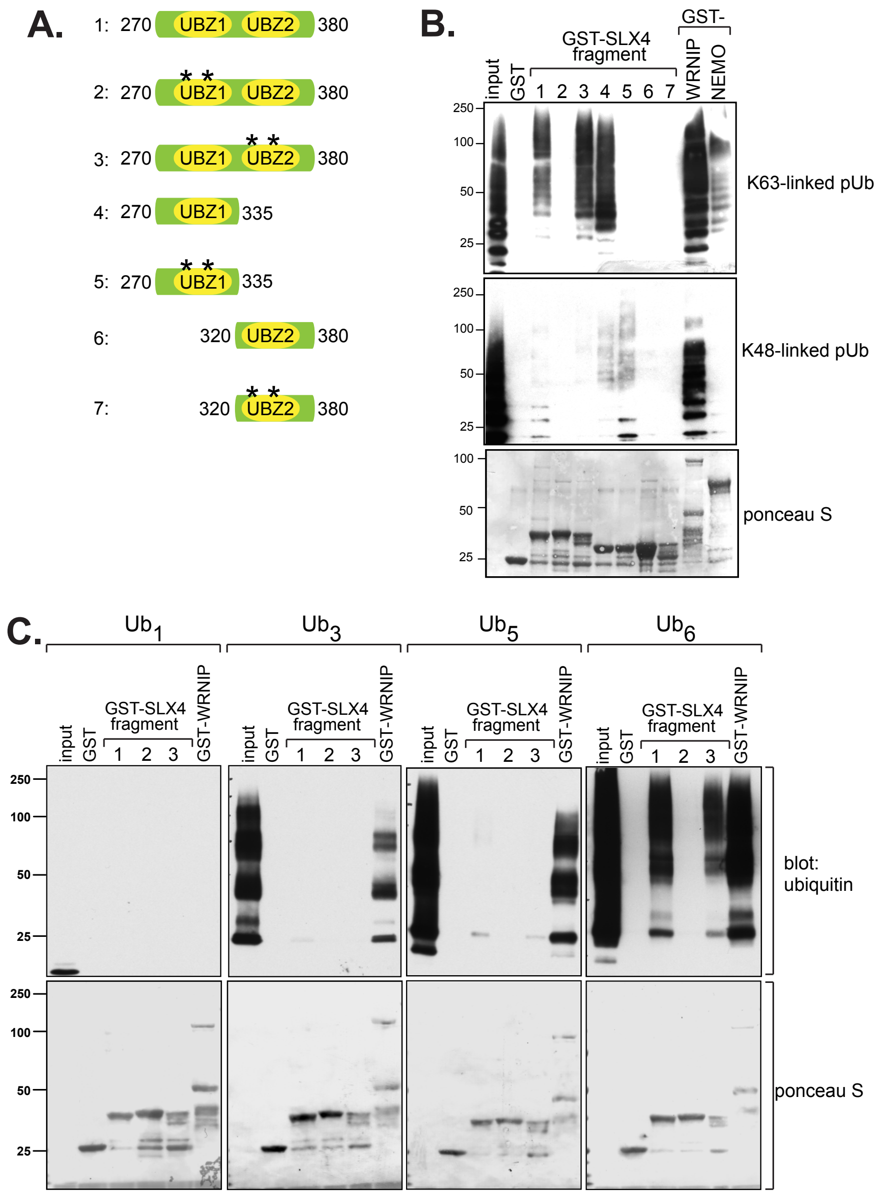

Figure S2 Testing the ability of GST-tagged SLX4 UBZ domains to bind ubiquitin
(**A**) Schematic diagram of the GST-tagged SLX4 fragments used in ubiquitin binding assays. Asterisks denote cysteine to alanine mutations at Cys 296+Cys 299 in UBZ-1 or Cys 336+Cys 339 in UBZ-2, respectively. (**B**) The GST-tagged fragments in (A) or GST alone were immobilizied on glutathione sepharose and incubated with K48-linked or K63-linked poly-ubiquitin (pUb) chains. Pulldowns were subjected to SDS-PAGE gel and immunoblotted with anti-ubiquitin antibodies, The bottom panel shows a Ponceau staining of the membrane prior to blotting. (**C**) Same as (B) except that mono-ubiquitin or K63-linked poly-ubiquitin chains of the indicated length were used.

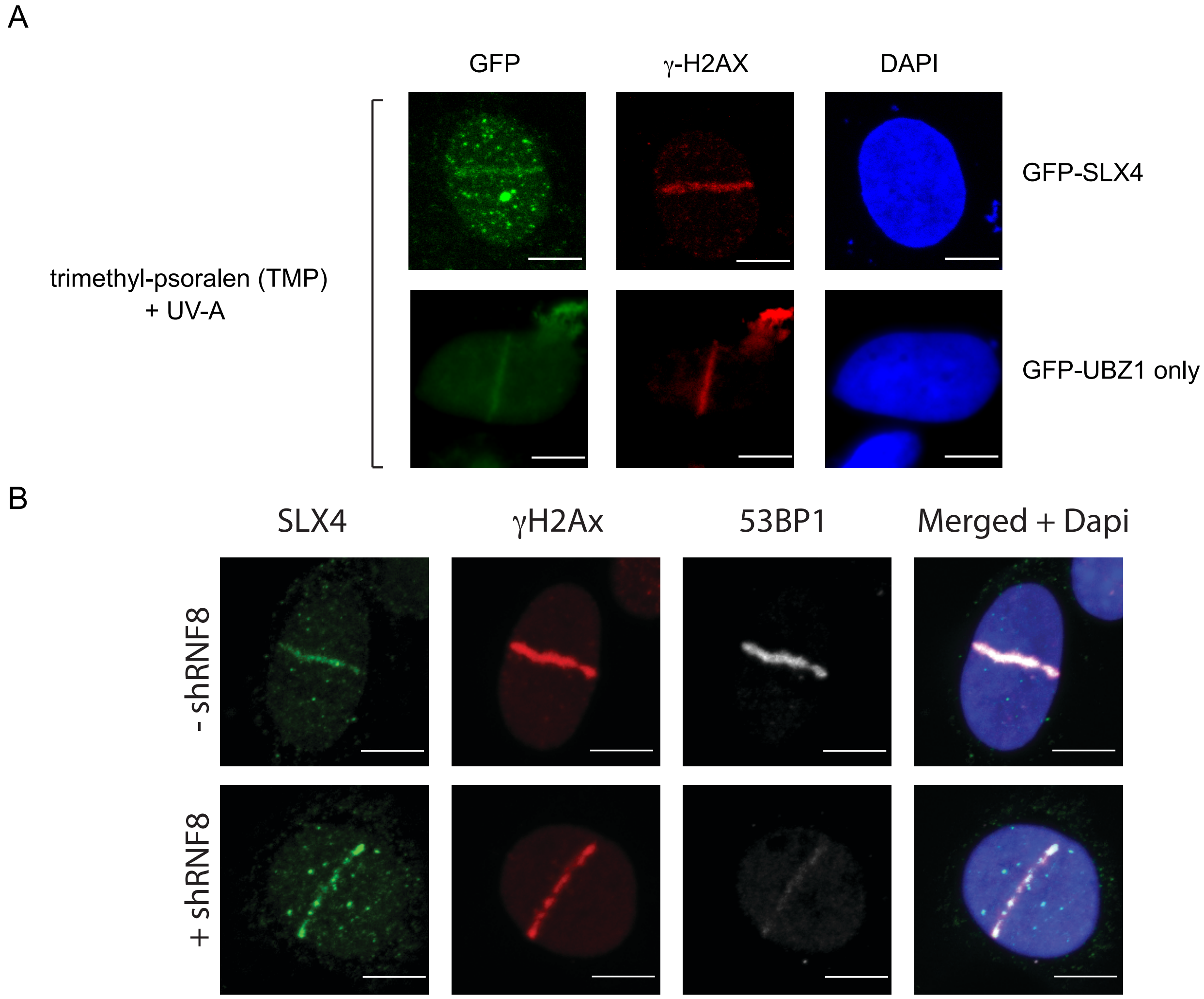

Figure S3. SLX4 recruitment to DNA damage sites

(**A**) U2OS cells stably expressing GFP-SLX4 or GFP-tagged UBZ1 were incubated with trimethyl-psoralen (TMP) and subjected to sub-nuclear micro-irradiation using a 355 nm UV-A laser. Cells were fixed and subjected to indirect immunofluorescence analysis with antibodies against GFP or γ–H2AX.

(B) U2OS cells stably expressing RNF8 shRNA were incubated (+shRNA8) or without (-shRNF8) with tetracycline for 24 h. Cells were then incubated with trimethyl-psoralen (TMP) and subjected to sub-nuclear micro-irradiation using a 355 nm UV-A laser. Cells were fixed and subjected to indirect immunofluorescence analysis with antibodies against GFP, γ–H2AX or 53BP1. Scale bars: 10µM

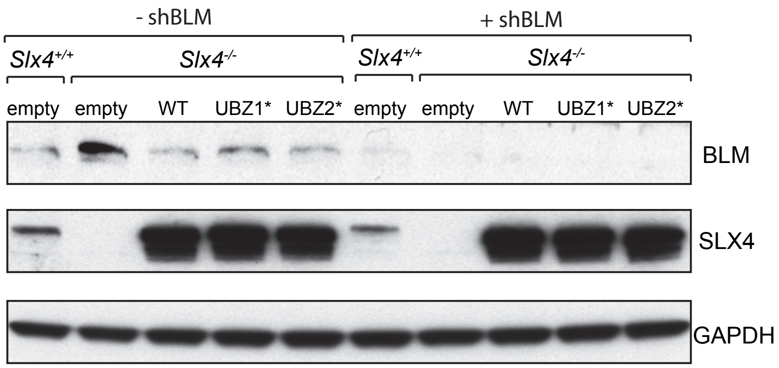

Figure S4. Depletion of BLM from MEFs using retrovirus-delivered shRNA
Western blot analysis of MEFs, of the genotypes indicated, infected with retroviruses expressing a BLM-specific shRNA (+) or with virus prepared with empty vector as control (-). WT - wild type.
